# Sources and levels of copper affect liver copper profile, intestinal morphology and cecal microbiota population of broiler chickens fed wheat-soybean meal diets

**DOI:** 10.1038/s41598-022-06204-9

**Published:** 2022-02-10

**Authors:** Hoai Thi Thanh Nguyen, Sarbast K. Kheravii, Shu-biao Wu, Julie R. Roberts, Robert A. Swick, Mehdi Toghyani

**Affiliations:** 1 Department of Animal Science, School of Environmental and Rural Science, University of New England, Armidale, NSW 2351 Australia; 2 Faculty of Science, School of Life and Environmental Sciences, The University of Sydney, Sydney, NSW 2006 Australia

**Keywords:** Physiology, Biotechnology, Animal biotechnology

## Abstract

Super dosing copper (Cu) has long been used as an alternative to antibiotic growth-promoters in broiler chickens’ diet to improve gut health. This study was designed to compare nutritional and growth-promoting levels of Cu hydroxychloride (CH) with CuSO_4_ on gut health bio-markers and liver mineral profile of broiler chickens. Ross 308 chicks (*n* = 864) were randomly assigned to eight treatments, as basal diet containing no supplemental Cu; the basal diet with 15 or 200 mg/kg Cu as CuSO_4_; or 15, 50, 100, 150 or 200 mg/kg Cu from CH. The highest liver Cu content was observed in birds fed the diets with 200 mg/kg CuSO_4_ (*P* < 0.01). Serum FITC-d concentration as the leaky gut marker, and liver malondialdehyde concentration were not affected. Copper level or source had no effect on cecal short chain fatty acid and the mRNA expression of five jejunal genes involved in gut integrity. Negative linear responses of Cu were observed on *Lactobacillus* (*P* = 0.032), *Bacteroides* (*P* = 0.033), and *Enterobacteriaceae* (*P* = 0.028) counts. The jejunal villus height increased in birds fed CH at 200 and 100 mg/kg (*P* < 0.05). Increasing Cu levels, linearly and quadratically (*P* < 0.001), increased Cu excretion.

## Introduction

Broiler chickens require copper (Cu) for optimum growth as Cu is an element with a very broad-spectrum action and can significantly birds’ overall health status^1^. However, toxic effects of Cu have been reported when it is overloaded^2,3^ on chicken health^4,5^, resulting in high Cu residues in poultry products^6^. Recommendation of Cu requirement for broilers by the National Research Council (NRC)^7^ is 8 mg/kg of finished feed, but Cu has been supplemented in broilers diet at far above nutritional levels (125 to 250 mg/kg), to improve growth performance as an alternative to antibiotic growth promoters^8^.

Copper super dosing has been shown to improve intestinal structure and function^9^ and alter the intestinal microbiota profile^10^. The beneficial effect of Cu supplementation on microflora in the alimentary tract through its bactericidal or bacteriostatic functions has been demonstrated in several studies^11–13^. Thereby it can reduce the susceptibility of birds to disease, reducing intestinal lymphocyte recruitment and infiltration and thus increasing nutrient absorption^12^. However, Gaetke and Chow^14^ showed that Cu toxicity can damage intestinal villi, reduced the ratio of villus height to crypt depth, and suppress the expression of tight junction proteins, negatively impacting on nutrient utilization. Chiou et al.^15^ reported that high Cu supplementation (500 mg/kg) in the diet damages duodenal villi and depresses feed intake, resulting in poor growth performance of broiler chickens. In addition, excessive intake of Cu may reduce the activities of various digestive enzymes in the intestine^16^.

Absorption and organ accumulation of Cu appear to be highly related to the solubility of the Cu source since a high Cu concentration in broiler diets may lead to increased fecal Cu^17,18^. According to Yang et al.^19^, a high level of Cu results in high Cu accumulation in the liver, and consequently, contributes to the increased Cu concentration of droppings, thereby leading to adverse effects on nutrient utilization. The source of Cu could significantly affect Cu concentration in the liver of broiler chickens^20^. It is believed that the best method to determine the bioavailability of Cu source is to directly measure its retention in the liver^21^.

Copper sulfate is the most commonly used source of Cu for poultry^8,22^. It is very soluble in both water and acidic solvents. Another Cu source being used and considered by poultry producers is Cu hydroxychloride (CH), a crystalline inorganic mineral source formed by covalent bonds between Cu and hydroxy groups. This form of Cu is non-hygroscopic and poorly soluble in water but highly soluble in acidic conditions^22,23^. Copper hydroxychloride has been shown to be more bioavailable to broilers because of its higher stability in feed and premixes and its lower binding activity with other dietary constituents^24^. Due to different relative bioavailability and solubility of Cu sulfate and CH, it might differently affect intestinal microbiota and tissue mineralization. Persson et al.^25^ demonstrated that high levels of Cu as Cu sulfate are known to interfere with phytate at intestinal pH, and the resulting complexes tend to be resistant to the hydrolytic activity of phytase.

The current study was designed to evaluate the effect of Cu sulfate versus CH on tissue mineralization and oxidation, intestinal morphology and integrity and cecal microbiota populations of broiler chickens, when fed at either nutritional (15 mg/kg) or growth-promoting levels (200 mg/kg). In addition, graded doses of CH at growth-promoting levels (50, 100 and 150 mg/kg) were tested. The data on birds’ performance parameters obtained from this study have already been published, suggesting that copper supplementation in the form of CH is more efficacious than Cu from sulfate in promoting growth performance, both at the nutritional and super-dosing levels^26^.

## Methods

All the experimental procedures of this study were reviewed and approved by the University of New England Animal Ethics Committee (AEC17-109). The study was performed in accordance and full compliance with the approved guidelines and regulations. Following the AVMA 2020 euthanasia guidelines, the sacrificed birds were first electrically stunned and sedated prior to euthanasia, and then decapitated using a sharp knife. The study reported in this manuscript follows the recommendations in ARRIVE guidelines.

### Birds and housing

A total number of 864 male day-old Ross 308 chicks were transported from Aviagen hatchery, (Goulburn, NSW, Australia) to the Centre for Animal Research and Teaching at the University of New England. Upon arrival, chicks were weighed and randomly assigned to one of 48 floor pens. Each pen measured 1.2 m × 0.75 m and was equipped with a tube feeder, two cup-drinkers and fresh hardwood shavings were used as bedding material. Room temperature was maintained at 33 ± 1.0 °C during the first three days and gradually reduced to 24 °C at the end of week 3. The lighting program and ventilation followed the recommendations set in the Ross 308 breed management manual^27^. Birds had ad libitum access to water and feed throughout the entire study.

### Experimental treatments and design

The birds were randomly assigned to eight dietary treatments, each replicated six times, with 18 chicks per replicate in floor pens. The dietary treatments met the nutrient specifications of the strain as recommended by the Ross 308 guidelines^27^ (Table 1). Eight mineral premixes containing different sources and levels of copper were also formulated and mixed. The eight dietary treatments consisted: (1)—basal diet (negative control diet—NC) containing no supplemental Cu; (2)—basal diet supplemented with 15 mg/kg Cu as CuSO_4_; (3)—basal diet supplemented with 200 mg/kg Cu as CuSO_4_; (4)—basal diet supplemented with 15 mg/kg Cu as CH; (5)—basal diet supplemented with 50 mg/kg Cu as CH; (6)—basal diet supplemented with 100 mg/kg Cu as CH; (7)—basal diet supplemented with 150 mg/kg Cu as CH and (8)—basal diet supplemented with 200 mg/kg Cu as CH (Selko IntelliBond Cu, Trouw Nutrition, Netherlands). The broiler chicks received the wheat-soybean meal based experimental diets in two phases from d 0 to 14 (starter phase) and d 14 to 35 (grower phase).

**Table 1:** Diet composition and nutritive value of the experimental diets (as-fed basis).

Ingredients (%)	Starter (d 1 to 14)	Grower (d 14 to 35)
Wheat	53.79	55.77
Soybean meal	27.71	19.85
Canola meal	9.74	12.80
Rice bran	4.73	6.29
Canola oil	1.56	3.07
Limestone	1.19	1.18
Dicalcium phosphate^a^	0.28	0.05
Salt	0.17	0.12
Na bicarb	0.10	0.13
Mineral premix^b^	0.10	0.10
Vitamin premix^c^	0.09	0.09
Choline Cl 60%	0.05	0.05
L-lysine HCl 78.4	0.23	0.24
D,L-methionine	0.19	0.17
L-threonine	0.04	0.05
Xylanase	0.02	0.02
Phytase	0.01	0.01
**Calculated nutrients**		
ME, kcal/kg	3000	3150
Crude protein, %	23.86	21.62
Crude fat, %	4.62	6.64
Crude fiber, %	3.62	3.85
d Arg, %	1.34	1.15
d Lys, %	1.24	1.10
d Met, %	0.53	0.48
d M + C, %	0.90	0.83
d Trp, %	0.28	0.25
d Ile, %	0.89	0.78
d Thr, %	0.79	0.72
d Val, %	0.98	0.88
Ca, %	0.85	0.80
Phosphorus available, %	0.43	0.40

^a^Dicalcium phosphate contained: phosphorus, 18%; calcium, 21%.

^b^The Cu-free trace mineral concentrate supplied per kilogram of diet: Zn (sulphate), 60 mg; Fe (sulfate), 40 mg; I (iodide), 1.5 mg; Se (selenate), 0.3 mg; Mn (sulfate), 80 mg; millrun-based carrier, 128 mg; mineral oil, 100 mg.

^c^Vitamin concentrate supplied per kilogram of diet: retinol, 12,000 IU; cholecalciferol, 5000 IU; tocopheryl acetate, 75 mg, menadione, 3 mg; thiamine, 3 mg; riboflavin, 8 mg; niacin, 55 mg; pantothenate, 13 mg; pyridoxine, 5 mg; folate, 2 mg; cyanocobalamin, 16 μg; biotin, 200 μg; cereal-based carrier, 149 mg; mineral oil, 2.5 mg.

### Data collection

Triple representative composite samples from all the diets and mineral premixes were collected and analyzed to measure Cu concentration, determined in duplicates (Table 4).

On day 14, to evaluate gut integrity, three birds per replicate were orally gavaged with 1 ml of fluorescein isothiocyanate-dextran solution (FITC-d), a marker for leaky gut evaluation (100 mg MW 4000, Sigma–Aldrich Co., Castle Hill, NSW, Australia). At 2.5 h after inoculation, the birds were electrically stunned and decapitated for blood collection. Blood samples were collected in vacutainer tubes (BD, Wokingham, Berkshire, UK) and then centrifuged at 3000× *g* for 10 min at 4 °C to separate the serum, and subsequently frozen at − 20 °C prior to analysis for FITC-d values.

The cecal content from the three sacrificed birds, on day 14, was collected into ice-cooled containers, and then subsequently frozen at − 20 °C for short-chain fatty acids (SCFAs) analysis. Sub-samples of cecal digesta were collected in Eppendorf tubes and directly snap-frozen in liquid nitrogen and kept at − 80 °C until analysed for microbial population by real-time quantitative PCR (qPCR).

A 1-cm section of jejunum from each bird was collected at the Meckel’s diverticulum. The jejunum sections were flushed with sterile ice-cold phosphate buffered saline solution (PBS; pH 7.4) and stored into 2 ml Eppendorf tubes with 1.5 ml RNAlater (Invitrogen, Thermo Fisher Scientific, California, USA), and then transferred into a − 80 °C freezer prior to gene expression analysis. Liver samples were taken from those three birds in each pen, then stored at − 20 °C prior to mineral analysis.

On d 35 post-hatch, three randomly representative birds were sacrificed from each pen to collect liver samples for mineral analysis. A sub-sample of the liver was collected and snap-frozen in liquid nitrogen to measure the malondialdehyde (MDA) levels. Then, mid jejunum sections were collected and washed with PBS, and then fixed in 10% buffered formalin until processing. Distal ileum content from the same birds was also collected to be analyzed for mineral concentration.

### Chemical analysis

#### Tissue mineral concentration analysis

The mineral content in premixes, diets, liver, and distal ileum contents were determined using an inductively coupled plasma-optical emission spectrometer (ICP-OES) (Agilent, Mulgrave, Victoria, Australia), following the method described by Nguyen et al.^26^.

Triplicate representative composite sample from each diet (as-fed basis) was collected and ground into fine particles (0.5 mm) to analyse Cu concentration in duplicate. Liver samples and distal ileum digesta were freeze-dried at − 50 °C and then ground to pass a 0.5 mm sieve. Then 0.5 g of each sample was weighed in white Teflon tubes (Milestone, Sorisole, Bergamo, Italy) and then digested in 1 ml distilled water and 4 mL concentrate HCl (70%) in an Ultra wave Microwave Digestion system (Milestone, Sorisole, Bergamo, Italy). The digested samples were diluted with distilled water to a volume of 25 ml in a 30 ml volumetric flask for analysis of mineral concentration by ICP-OES instrument.

#### Liver lipid peroxidation

The extent of lipid peroxidation of liver samples was measured using a lipid peroxidation assay kit (Abcam, Cambridge, UK), following the manufacturer’s instructions. The amount of MDA in the liver sample was quantified using a calibration curve developed with the MDA standard solution, following the method described by Kurantowicz et al.^28^.

#### Serum FITC-d measurement

The serum concentration of FITC-d was quantified according to the method described by Kuttappan et al.^29^ Briefly, serum samples were diluted 1:1 with PBS for the assay and black 96-well plates were used to prevent crosstalk between samples. The concentration of FITC-d per ml of serum was measured at an excitation wavelength of 485 nm and an emission wavelength of 528 nm using a black microplate reader (SpectraMax M2e, Molecular Devices, San Jose, California, USA). Levels of fluorescence in the samples were converted to respective FITC-d µg per mL of serum based on a calculated standard curve.

#### Cecal SCFAs analysis

Cecal concentrations of SCFAs were measured according to the method described by Jensen et al.^30^ with minor modifications. Briefly, 0.8 g of cecal digesta was weighed into centrifuge tubes and 1 ml of 0.01 M ethylbutyric acid (internal standard) solution added. The solution was vortexed and centrifuged at 15,000× *g* at 5 °C for 20 min. 1 ml supernatant was transferred to 8 ml vials (placed on ice), then 2.5 ml of ether and 0.5 ml of concentrated HCl (36%) were added. The solution was vortexed for one min, then centrifuged at 3000× *g* for 15 min in 5 °C; 400 µl of the resulting supernatant was transferred into 2 ml gas chromatograph vials and mixed with 40 µl N-tert-butyl-dimethylsilyl-N-methyltrifluoroacetamide. This solution was vortexed and heated at 80 °C in a heating block for 20 min and then left at room temperature for at least 48 h. Then 0.5 ml ether was added into the gas chromatograph vials before analysis using a Varian CP3400 CX gas Chromatograph (Varian Analytical Instruments, Palo Alto, CA, USA). The value of SCFAs is expressed as μmol/g wet cecal digesta.

#### Jejunal morphology

The morphological analysis followed the method described by M’Sadeq et al.^31^. Jejunum samples were fixed in 10% neutral buffered formalin and prepared using paraffin embedding techniques. Samples were processed in Leica TP1,020 45 processor (GMI Inc., Ramsey, MN) according to the program as follows: 30% ethanol for 2 h; 50% ethanol for 2 h; 70% ethanol for 2 h; 80% ethanol for 2 h; 95% ethanol for 1 h; absolute ethanol for 1 h; absolute ethanol for 1 h; 50:50 ethanol:xylol for 1 h; xylol for 1 h; xylol for 1 h; paraplast for 2 h and paraplast 1 Vac for 2 h. A microtome (Leitz 1516; Leica Microsystems, Bensheim, D-64625, German) was used to make 5-µm cuts and mounted on glass slides and then stained using Harris’s hematoxylin and eosin staining method. The specimens were examined via a light microscope (Olympus CX41 microscope) and images were captured with the software Analysis 5.0 (Olympus Soft Imaging Solutions GmbH, Munster, Germany). Sixteen villus height and crypt depth measurements per treatment were taken and averaged to provide equal morphological representation.

#### Quantification of cecal bacterial groups

Cecal bacterial DNA extraction was performed following the method described by Kheravii et al.^32^. In brief, 65 mg of frozen cecal samples were added to 300 mg of glass beads. QIAxtractor DNA Reagents and QIAxtractor DNA plasticware kits (Qiagen, Inc., Doncaster, VIC, Australia) were used for DNA extraction. Then samples were lysed with 300 µL of Qiagen Lysis Buffer, with cells disrupted by shaking the tubes in a bead beater mill (Retsch GmbH & Co, Haan, Germany). Samples were then placed in a heating block for 2 h at 55 °C followed by centrifugation at 20,000× *g* for 5 min. Then the DNA was extracted using an X-tractor gene automated DNA extraction system (Corbett Life Science, Sydney, Australia). The extracted DNA samples were checked for quantity and purity on a NanoDrop ND-8000 spectrophotometer (Thermo Fisher Scientific, Waltham, USA). DNA with ratios of A260/A280 being > 1.8 were considered of high purity and were stored at − 20 °C.

The extracted cecal DNA was diluted 20 times in nuclease-free water, and the quantitative real-time polymerase chain reaction (PCR) was performed to quantify seven bacterial groups with a real-time PCR system Rotorgene 6000 (Corbett, Sydney, Australia). The PCR was performed in duplicate for each sample. A SYBRGreen containing Mix (SensiMix SYBR No-Rox, Bioline, Sydney, Australia) was applied for all groups of bacteria to quantify Total bacteria, *Bacillus*, *Bacteroides*, *Bifidobacterium*, *Lactobacillus*, *Ruminococcus*, and *Enterobacteria*. The primers used for these microbial populations are shown in Table 2. Bacteria numbers were expressed as log_10_ (genomic DNA copy number)/g wet digesta.

**Table 2:** Primers sequences use for the qPCR analysis of selected bacteria groups.

Target group	Primer sequences (5′–3′)	Annealing temp. (°C)	References
*Bacillus *spp.	F-GCA ACG AGC GCA ACC CTT GA R-TCA TCC CCA CCT TCC CC GGT	63	^33^
*Bacteroides *spp.	F-GAG AGG AAG GTC CCC CAC R-CGC TAC TTG GCT GGT TCA G	63	^34^
*Bifidobacterium *spp.	F-GCG TCC GCT GTG GGC R-CTT CTC CGG CAT GGT GTT G	63	^35^
*Enterobacteriaceae*	F-CAT TGA CGT TAC CCG CAG AAG AAG C R-CTC TAC GAG ACT CAA GCT TGC	63	^36^
*Lactobacillus *spp.	F-CAC CGC TAC ACA TGG AG R-AGC AGT AGG GAA TCT TCC A	63	^37^
*Ruminococcus *spp.	F-GGC GGC YTR CTG GGC TTT R-CCA GGT GGA TWA CTT ATT GTG TTA A	63	^38^
Total bacteria	F-CGG YCC AGA CTC CTA CGG G R-TTA CCG CGG CTG CTG GCA C	63	^39^

#### Jejunal gene expression analysis

Jejunal gene expression was measured with slight modifications following the method used by Kheravii et al.^40^. Briefly, total RNA from approximately 80 mg of jejunal tissues was extracted after homogenization in TRIsure™ (Bioline, Sydney, Australia), following the manufacturer’s instructions. Total RNA of each sample was purified using ISOLATE II RNA Mini Kit (Bioline, Sydney, Australia) as per the manufacturer’s instructions. The quantity and quality of total RNA were determined using a NanoDrop ND-8000 spectrophotometer (Thermo Fisher Scientific, Waltham, USA). RNA integrity number (RIN) was evaluated with an Agilent 2100 Bioanalyzer (Agilent Technologies, Inc., Waldbronn, Germany) using RNA 6000 Nano kit. RNA samples were considered of high quality for downstream analysis if the RIN value was greater than 7.5^41^.

The extracted RNA of each sample was reverse transcribed into cDNA using the SensiFAST cDNA Synthesis Kit as per the manufacturer’s instructions. The cDNA was diluted 10 times with nuclease-free water and stored at – 20 °C for further analysis.

Quantitative PCR was performed using a SYBR Green kit SensiFAST™ SYBR® No-ROX (Bioline, Sydney, Australia) with Rotorgene 6000 real-time PCR machine (Corbett Research, Sydney, Australia). The geNorm module in qbase + software was employed to determine two most stable genes among eight different reference genes, 18S, ACTB, GAPDH, HPRT1, HMBS, TBP, SDHA, and YWHAZ. Based on the expression stability, GAPDH and TBP were used to normalize the target genes in the jejunum. The primers of the selected genes are described in Table 3.

**Table 3:** Primers used for quantitative real-time PCR.

Target genes	Gene name	Primer sequence (5′–3′)	Ta	Size (bp)	References
CLDN1	Claudin 1	F-CTTCATCATTGCAGGTCTGTCAG R-AAATCTGGTGTTAACGGGTGTG	60	103	^42^
CLDN5	Claudin 5	F-GCAGGTCGCCAGAGATACAG R-CCACGAAGCCTCTCATAGCC	60	162	^42^
JAM2	Junctional adhesion molecule 2	F-AGACAGGAACAGGCAGTGCTAG R-ATCCAATCCCATTTGAGGCTAC	60	135	^42^
OCLD	Occludin	F-ACGGCAGCACCTACCTCAA R-GGGCGAAGAAGCAGATGAG	60	123	^43^
TJP1	Tight junction protein 1	F-GGATGTTTATTTGGGCGGC R-GTCACCGTGTGTTGTTCCCAT	60	187	^42^
GAPDH	Glyceraldehyde-3-Phosphate Dehydrogenase	F-GAAGCTTACTGGAATGGCTTTCC R-CGGCAGGTCAGGTCAACAA	60	66	^44^
TBP	TATA-Box Binding Protein	F-TAGCCCGATGATGCCGTAT R-GTTCCCTGTGTCGCTTGC	61	147	^45^

### Statistical analysis

All the data derived were checked for normal distribution prior to conducting statistical analysis. Data were then subjected to one-way ANOVA analysis as a completely randomized design, using the General Linear Model procedure of SAS 9.3 package^46^. Every single pen was considered as an experimental unit, and the values presented are means with pooled standard error of the mean (SEM) (*n* = 48). Tukey’s HSD test was used to make pairwise comparisons between means. Significant values are based on *P* < 0.05; *P* > 0.05 and *P* < 0.10 are discussed if data suggested a trend. The linearity of responses to dietary Cu levels was established using linear and quadratic regression, and the associated *P* values presented.

## Results

The analyzed Cu concentration from the basal diet was 9.1 mg/kg for the starter phase and 7.6 mg/kg for the grower phase. The Cu contents of the other diets were very close to the calculated values (Table 4).

**Table 4:** The analyzed Cu concentration in the dietary treatments.

Treatments	Cu source	Cu added (mg/kg)	Calculated Cu (mg/kg)		Analyzed Cu (mg/kg)	
			Starter	Grower	Starter	Grower
1—NC	None	0	8	8	9.1	7.6
2	CuSO_4_	15	23	23	27.3	24.8
3	CuSO_4_	200	208	208	223.2	222.4
4	CH	15	23	23	26.1	25.6
5	CH	50	58	58	68.7	59.7
6	CH	100	108	108	116.5	110.3
7	CH	150	158	158	164.0	165.2
8	CH	200	208	208	215.3	230.3

Values based on chemical analysis of duplicate samples of diets and reported on an as-fed basis. *NC* Negative control, no added Cu; *CH* Copper hydroxychloride (Intellibond Cu).

The dietary treatments significantly influenced liver Cu concentration of broilers both at d 14 and 35 (*P* < 0.01; Table 5). Increased hepatic Cu content was observed with increasing supplemental Cu at d 14 and 35 both linearly and quadratically (*P* < 0.01). Birds fed 200 mg/kg Cu from CuSO_4_ accumulated higher Cu in the liver than those given the NC diet and other Cu supplemented diets, except for birds fed 200 mg/kg Cu as CH at d 35. The accumulation of Zn, Fe, P and Ca in the liver was not affected by the dietary treatments (*P* > 0.05). The source and level of supplemental Cu did not influence on MDA values in the liver at d 35 (*P* > 0.05; Table 5).

**Table 5:** Liver mineral concentrations and MDA value of broiler chickens fed the dietary treatments.

Treatments^c^	Cu (µg/g)		Zn (µg/g)		Fe (µg/g)		Ca (µg/g)		MDA (nmol/mg)
	D 14	D 35	D 14	D35	D 14	D 35	D 14	D 35	D 35
NC	12.1^b^	12.7^b^	80.2	99.4	406	646	169	182	0.533
CuSO_4_ 15 mg/kg	13.5^ab^	12.9^b^	87.9	87.5	423	649	157	203	1.187
CuSO_4_ 200 mg/kg	15.6^a^	16.4^a^	85.7	96.0	518	689	170	187	0.937
CH 15 mg/kg	12.3^b^	11.9^b^	84.4	94.1	411	556	177	214	0.757
CH 50 mg/kg	12.6^b^	11.6^b^	85.6	93.3	415	616	177	278	0.654
CH 100 mg/kg	12.6^b^	12.7^b^	84.4	98.3	391	617	181	222	0.833
CH 150 mg/kg	12.9^b^	13.8^b^	81.5	93.6	406	676	170	272	0.659
CH 200 mg/kg	14.3^ab^	14.6^ab^	82.8	94.1	392	593	163	220	0.849
SEM	0.496	1.104	2.21	5.79	45.3	46.68	11.18	42.65	0.265
***P*-values**									
ANOVA GLM	0.001	0.009	0.292	0.903	0.580	0.540	0.836	0.672	0.767
Linear	0.001	0.003	0.791	0.829	0.393	0.377	0.877	0.799	0.776
Quadratic	0.002	0.007	0.901	0.976	0.680	0.679	0.522	0.359	0.918

^a–b^Values in a column with no common superscripts differ significantly (*P* ≤ 0.05). Mean values are based on 3 birds per replicate and 6 replicates per treatment.

^c^*NC* Negative control, no added Cu; *CH* Copper hydroxychloride (Intellibond Cu). *Cu* Copper, *Zn* Zinc, *Fe* Iron, *Ca* Calcium, *MDA* Malondialdehyde.

**Figure 1:**
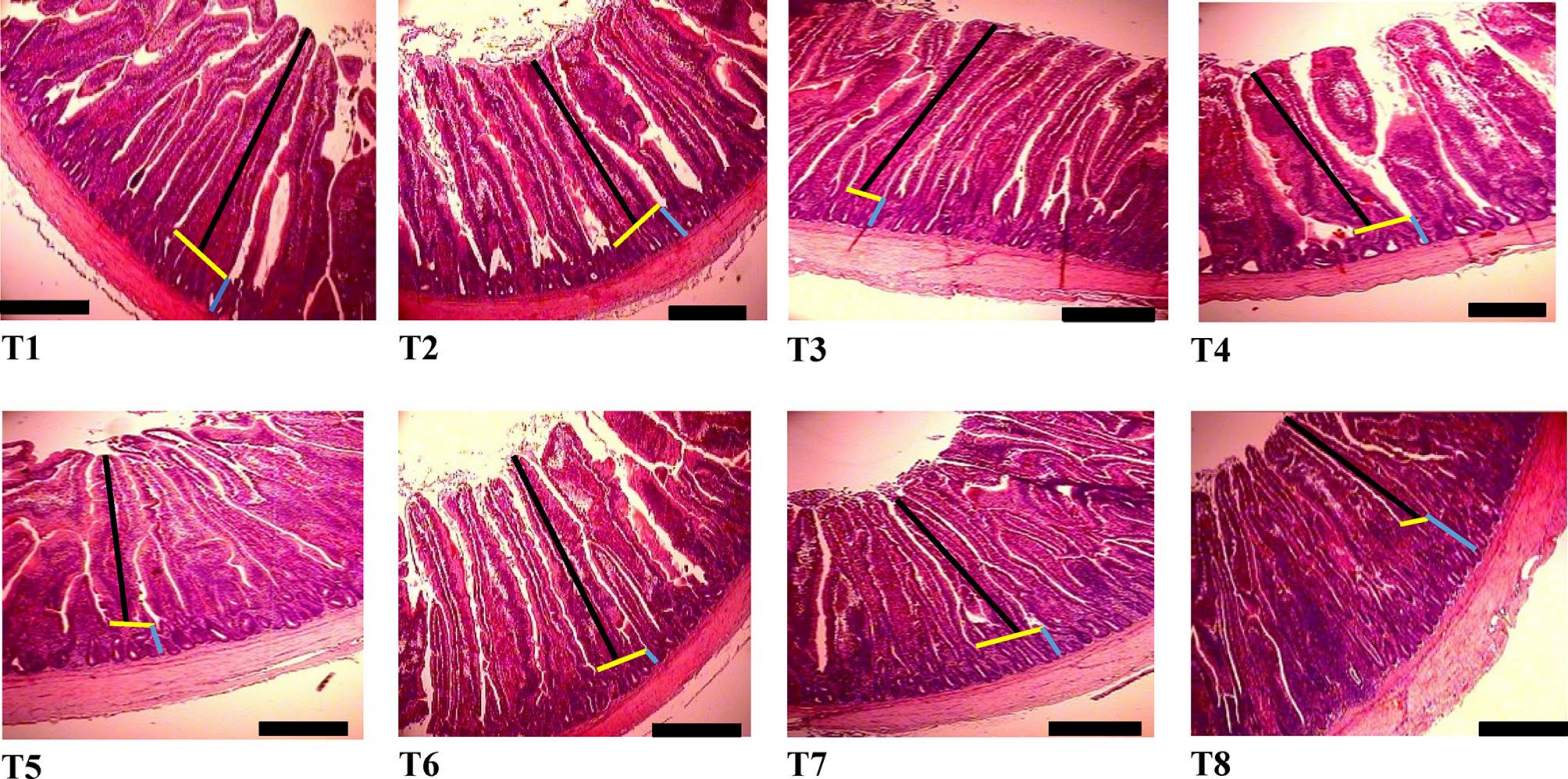
Histological representation of the jejunum in broilers (scale bar: 100 µm). Villus height (black line) was measured from the tip of the villi to the villus-crypt junction (yellow line); Crypt depth (blue line) was measured on the depth of invagination between adjacent villi. T1: NC diet, containing no supplemental Cu; T2: containing 15 mg/kg Cu as CuSO_4_; T3: containing 200 mg/kg Cu as CuSO_4_; T4: containing 15 mg/kg Cu as Copper hydroxychloride; T5: containing 50 mg/kg Cu as Copper hydroxychloride; T6: containing 100 mg/kg Cu as Copper hydroxychloride; T7: containing 150 mg/kg Cu as Copper hydroxychloride; T8: containing 200 mg/kg Cu as Copper hydroxychloride.

According to the data presented in Table 6, jejunal villus height at d 14 was significantly affected by the source and inclusion level of supplemental Cu (*P* < 0.01) but no linear or quadratic responses were observed (*P* > 0.05). As illustrated in Fig. 1, birds receiving the diet with 200 mg/kg Cu as CH had higher villus height in jejunum section compared to those ingested 200 mg/kg CuSO_4_ supplemented diet but similar to birds fed the NC diet. However, crypt depth and villus height to crypt depth ratio were not significantly affected as a result of supplemental Cu (*P* > 0.05).

**Table 6:** Histomorphological measurements in jejunum of broilers on day 14.

Treatments^c^	Crypt depth (µm)	Villus height (µm)	Villus height: crypt depth
NC	245	1212^ab^	5.05
CuSO_4_ 15 mg/kg	296	1185^b^	4.04
CuSO_4_ 200 mg/kg	286	1134^b^	4.38
CH 15 mg/kg	267	1159^b^	4.43
CH 50 mg/kg	269	1277^ab^	4.83
CH 100 mg/kg	266	1309^a^	5.02
CH 150 mg/kg	289	1169^b^	4.18
CH 200 mg/kg	304	1386^a^	4.85
SEM	23.28	42.80	0.439
***P*-values**			
ANOVA GLM	0.676	0.001	0.606
Linear	0.159	0.295	0.895
Quadratic	0.368	0.415	0.978

^a–b^Values in a column with no common superscripts differ significantly (*P* ≤ 0.05). Mean values are based on 3 birds per replicate and 6 replicates per treatment.

^c^*NC* Negative control, no added Cu, *CH* Copper hydroxychloride (Intellibond Cu).

The mineral composition of distal ileum content collected at d 35 is presented in Table 7. Distal ileum Cu concentration increased both linearly and quadratically as dietary Cu concentration increased (*P* < 0.01). Corresponding to the dietary Cu levels, distal ileum Cu content was the highest at 200 mg/kg Cu either from CuSO_4_ or CH. Distal ileum Fe content increased linearly and quadratically in response to Cu levels of the diet (*P* < 0.05). Broilers fed the NC and the diet with CuSO_4_ at 200 mg/kg tended (*P* = 0.084) to excrete the lowest and the highest Fe, respectively. Numerically higher Mn, Zn, Ca and P content were observed in distal ileum content from birds fed CuSO_4_ at 200 mg/kg, though the differences did not reach statistical significance (*P* > 0.05). Table 7 also shows serum FITC-d value determined at d 14 which was not significantly affected by the dietary treatments (*P* > 0.05).

**Table 7:** Mineral concentration in distal ileum content at day 35 and serum FITC-d values at day 14.

Treatments^g^	Distal ileum						FITC-d (µg/ml)
	Cu, µg/g	Fe, µg/g	Zn, µg/g	Mn, µg/g	Ca, g/kg	P, g/kg	
NC	48^f^	392	318	492	1.18	0.80	0.068
CuSO_4_ 15 mg/kg	98^ef^	431	368	550	1.42	0.90	0.096
CuSO_4_ 200 mg/kg	995^a^	504	388	598	1.50	0.99	0.121
CH 15 mg/kg	99^ef^	448	318	537	1.25	0.84	0.133
CH 50 mg/kg	300^de^	477	336	558	1.29	0.85	0.094
CH 100 mg/kg	513^cd^	422	326	494	1.20	0.84	0.085
CH 150 mg/kg	621^bc^	482	334	537	1.27	0.81	0.081
CH 200 mg/kg	821^b^	478	334	523	1.35	0.83	0.101
SEM	47.5	26.6	20.6	31.4	0.093	0.046	0.021
***P*-values**							
ANOVA GLM	0.001	0.084	0.214	0.314	0.244	0.146	0.376
Linear	0.001	0.008	0.231	0.323	0.170	0.281	0.654
Quadratic	0.001	0.014	0.357	0.502	0.453	0.398	0.684

^a–f^Values in a column with no common superscripts differ significantly (P ≤ 0.05). Mean values are based on 3 birds per replicate and 6 replicates per treatment.

^g^*NC* Negative control, no added Cu, *CH* Copper hydroxychloride (Intellibond Cu). *Cu* Copper, *Fe* Iron, *Zn* Zinc, *Mn* Manganese, *Ca* Calcium, *P* Phosphorus, *FITC-d* fluorescein isothiocyanate-dextran.

The influence of dietary treatments on cecal microbiota of broilers at d 14 is presented in Table 8. There was a linear (*P* = 0.032) response of supplemental Cu on *Lactobacillus* count, where increasing Cu inclusion in the diet decreased the number of *Lactobacillus*. *Bacteroides* and *Enterobacteriaceae* counts were also linearly affected (*P* = 0.033; *P* = 0.028, respectively) by Cu content of the feed, where higher Cu supplementation resulted in lower counts of these bacterial groups. Higher supplemental Cu in the diet tended (*P* = 0.082) to linearly decrease total bacteria load.

**Table 8:** Bacterial composition (Log_10_ copy numbers g^−1^) in ceca content of broiler chickens at day 14.

Treatments^a^	Lacto-bacillus	Rumino-coccus	Bacter-oides	Bacillus	Bifido-bacterium	Entero-bacteriaceae	Total bacteria
NC	9.07	9.06	5.71	7.65	9.64	9.43	11.21
CuSO_4_ 15 mg/kg	8.91	8.93	5.63	7.62	9.88	8.98	11.10
CuSO_4_ 200 mg/kg	8.59	8.84	5.64	7.46	9.88	8.60	10.98
CH 15 mg/kg	8.81	8.92	5.61	7.32	9.75	9.18	11.06
CH 50 mg/kg	9.04	9.11	5.65	7.24	9.36	8.93	11.12
CH 100 mg/kg	8.84	9.18	5.59	7.49	9.69	8.91	11.11
CH 150 mg/kg	8.96	9.06	5.53	7.33	9.65	9.10	11.07
CH 200 mg/kg	8.52	8.85	5.44	6.79	9.78	8.49	10.85
SEM	0.181	0.148	0.065	0.278	0.406	0.381	0.104
***P*-values**							
ANOVA GLM	0.316	0.646	0.171	0.474	0.990	0.730	0.388
Linear	0.032	0.391	0.033	0.143	0.753	0.028	0.082
Quadratic	0.055	0.130	0.100	0.345	0.802	0.067	0.222

Mean values are based on 3 birds per replicate and 6 replicates per treatment.

^a^*NC* Negative control, no added Cu, *CH* Copper hydroxychloride (Intellibond Cu).

According to the data presented in Table 9, the concentration of SCFAs measured in ceca content of birds at d 14 was not significantly affected by the dietary treatments (*P* > 0.05). However, propionic acid tended to (*P* = 0.061) increase quadratically in response to increasing dietary Cu.

**Table 9:** Concentration of short chain fatty acids (μmol g^−1^ digesta) in ceca content of broiler chickens on day 14.

Treatments^a^	Acetic	Propionic	Iso-Butyric	Butyric	Iso-Valeric	Valeric	Lactic	Succinic	Total
NC	89	3.84	0.57	25	6.39	0.87	1.29	23	152
CuSO_4_ 15 mg/kg	86	3.41	0.46	24	7.24	0.56	1.55	22	145
CuSO_4_ 200 mg/kg	101	4.17	0.53	26	7.25	0.74	1.25	40	175
CH 15 mg/kg	103	3.77	0.49	26	7.90	0.64	1.54	37	180
CH 50 mg/kg	111	4.20	0.72	32	7.98	1.02	1.33	36	194
CH 100 mg/kg	111	3.96	0.65	30	7.21	0.98	1.63	34	189
CH 150 mg/kg	91	5.12	0.59	29	9.34	0.84	1.54	33	171
CH 200 mg/kg	101	4.17	0.66	25	9.36	0.72	1.67	36	178
SEM	8.44	0.492	0.075	3.36	1.22	0.116	0.250	5.59	14.07
***P*-values**									
ANOVA GLM	0.314	0.429	0.208	0.655	0.638	0.103	0.845	0.257	0.214
Linear	0.560	0.113	0.347	0.977	0.188	0.906	0.777	0.064	0.291
Quadratic	0.342	0.061	0.137	0.434	0.378	0.326	0.633	0.162	0.144

Mean values are based on 3 birds per replicate and 6 replicates per treatment.

^a^*NC* Negative control, no added Cu, *CH* Copper hydroxychloride (Intellibond Cu).

There was no significant effect of dietary treatments on the mRNA expression of five jejunal tight junction genes involved in gut integrity of broilers at d 14 (*P* > 0.05; Table 10).

**Table 10:** Effect of dietary treatments on expression of jejunal tight junction genes^a^.

Treatments^a^	CLDN1	CLDN5	JAM2	OCLD	TJP1
NC	0.99	0.91	1.04	1.18	1.03
CuSO_4_ 15 mg/kg	1.17	1.06	1.02	0.96	0.98
CuSO_4_ 200 mg/kg	1.07	1.06	1.18	0.97	1.18
CH 15 mg/kg	0.92	0.93	1.04	0.99	1.17
CH 50 mg/kg	0.91	1.27	1.13	0.99	1.25
CH 100 mg/kg	1.16	0.99	0.89	1.06	0.97
CH 150 mg/kg	0.93	1.02	1.01	0.94	0.89
CH 200 mg/kg	1.15	1.22	1.12	1.08	0.88
SEM	0.13	0.15	0.16	0.11	0.18
***P*-values**					
ANOVA GLM	0.601	0.706	0.927	0.831	0.789
Linear	0.491	0.397	0.585	0.680	0.519
Quadratic	0.753	0.675	0.545	0.744	0.804

Mean values are based on 3 birds per replicate and 6 replicates per treatment.

^a^*NC* Negative control, no added Cu, *CH* Copper hydroxychloride (Intellibond Cu), *CLDN1* Claudin1, *CLDN5* Claudin5, *JAM2* Junctional adhesion molecule B, *OCLD* Occludin, *TJP1* Tight junction protein-1.

## Discussion

Analysis of the basal diet (negative control) showed that the analyzed Cu concentration was quite close to the calculated values. The Cu content of the NC diet was 9.1 and 7.6 mg/kg, in starter and grower diet, respectively, which was in line with NRC^7^ recommendation for Cu requirements for broilers.

Liver is an important organ for detoxification, metabolism, hormone synthesis, and secretion, immune responses, and for glycogen and trace minerals storage^47^. Therefore, liver health is directly related to the overall health and growth performance of broiler chickens. The liver has been reported to be the major site for Cu metabolism^15^ and a good indicator of body Cu status and relative bioavailability between sources^48,49^. Liver Cu concentration is influenced by variations in dietary Cu sources and concentrations^20^. Increased hepatic Cu content by increasing dietary Cu levels has been reported in broilers^21,50–54^, in cockerels^49^, and other animals^55,56^. Similarly, the results of the current study, showed that liver Cu accumulation increased with the increment of supplemental Cu. At the nutritional requirement level (15 mg/kg), the accumulation of Cu in the liver of the birds fed on diets containing added CH or CuSO_4_ was similar, attesting to the fact that the bioavailability of CH was the same as that of CuSO_4_. A reduction in the dietary supply of minerals is usually overcome by a combination of responses, including enhanced absorption, enhanced release from storage deposits, and reduced excretion^57^. Previous studies have reported a higher liver Cu content measured in birds fed diets with CH compared to those with CuSO_4_ at a similar level (250 mg/kg)^58^, or pigs (225 mg/kg)^59^, where CH was reported to have higher bioavailability compared to CuSO_4_. However, in the current study the increase in liver Cu content was markedly greater for the birds fed the diets supplemented with 200 mg/kg CuSO_4_ followed by 200 mg/kg CH. This contradiction might be due to the higher solubility of CuSO_4_ in comparison to CH, or the differences in homeostatic mechanisms including absorption, transport, storage, and exertion of Cu between the two sources, especially at the super-dosing levels.

Copper is an essential component of superoxide dismutase, an important enzyme in the antioxidant defense system, but Cu can also act as a pro-oxidant and promote oxidation particularly when used at excess levels^60^. Copper ions are actively oxidized and reduced, and may catalyze the formation of hydroxy radicals, which can lead to lipid peroxidation^61^. The findings of this study showed that feeding Cu up to 200 mg Cu/kg diet from CH or CuSO_4_ do not promote oxidative stress in the liver as the MDA values were not significantly affected by the dietary treatments, although numerically lower in birds fed the NC diet.

Copper absorption primarily occurs in the duodenum and upper jejunum^62^. Therefore, a minute amount of Cu would be absorbed in the ileum and the Cu concentration in distal ileum may indicate possible excreted Cu content. In this current study, increasing dietary Cu indeed increased Cu excretion, with the highest value measured for birds fed CuSO_4_ at 200 mg/kg. The highest distal ileum Cu content in this group could be linked to a reduced Cu retention in the bird body. The lower distal ileum Cu concentration in 200 mg/kg CH group compared to 200 mg/kg CuSO_4_ group; could be due to the stronger bonding in CH which allows Cu to become soluble gradually and more readily absorbed throughout the small intestine as Cu ions disperse in digesta contents^63^. This may suggest that supplementation with CH at the same level (200 mg/kg) could decrease the excretion of Cu, in comparison to CuSO_4_. The results obtained in this study also show that broilers fed the diets supplemented with 200 mg/kg Cu as CuSO_4_ tended to excrete the highest Fe; indicating less Fe was apparently absorbed due to potential interference of high dose of CuSO_4_ with Fe absorption in the upper gastro-intestine^64^, leading to reduced Fe bioavailability. Nonetheless, the complexity of endogenous trace mineral excretion in birds possibly impact their absorption and excretion; thus, further research is needed to confirm this.

Intestinal health problems are a significant issue in the poultry industry, especially in the context of pathogenic agents developing resistance to some antibiotics due to the continuous use of antibiotics as growth promoters. Disturbances of intestinal health because of genetic selection for high growth rate, high stocking density, poor ventilation, etc. are common in broiler chickens, which can hamper growth performance in particular feed efficiency due to inefficient digestion and absorption of nutrients. The morphology of the villi and crypts will reflect their functions, secretion of digestive enzymes and absorption of nutrients^15^. Intestine villi morphology is related to the intestine function and growth rate of the intestine. Longer intestinal villus mean a greater surface area for nutrient absorption^65^. Thus, poor gut health in broilers has indeed been associated with intestinal villus shortening^66^. Broiler performance and intestinal physiology can be positively affected by dietary Cu source since Cu directly stimulates villus regeneration in birds and can indirectly impact intestinal morphology through affecting the gut microbiota population^15^. Otto and Carlos^58^ found that Cu supplementation resulted in higher villus height in jejunum compared to the non-supplemental Cu treatment. However, the results obtained in this study suggest that a diet without any supplemental Cu (NC) does not negatively impact jejunal morphology of birds, possibly because the Cu contribution from the background ingredients (9.1 and 7.6 mg/kg in starter and grower diets, respectively) in the NC diet was adequate to support normal intestinal villi growth. Similarly, Fry et al.^60^ reported that villus height in the proximal jejunum of pigs fed CH (225 mg/kg) was similar to that of pigs fed no supplemental Cu. A stimulating effect of Cu on villus height was only detected when Cu was added at either level of 100 mg/kg or 200 mg/kg as CH. Birds fed Cu supplemented diets from CH had longer villi compared with CuSO_4_ supplementation at the same level (200 mg/kg), reflecting better absorption of nutrients in the intestine. Such results align with the enhanced feed efficiency observed in birds fed CH at 200 mg/kg compared with CuSO_4_ at the same level as reported in a previous publication from this study^26^.

Since different sources of Cu have different solubility, they may affect intestinal microbiota differently. High dietary Cu affected intestinal microbiota profile^11^, but it may cause some problems such as the development of Cu-resistant bacteria. Pang et al.^67^ observed that the high inclusion of Cu (187.5 mg/kg) from either Cu sulfate or CH did not influence the number of ileal *Lactobacillus* in broiler chickens. Leeson et al.^18^ reported no effect of additional supplemental Cu on cecal microbial counts in growing turkeys. When Cu was supplemented to the diets in this study, however, linear responses to Cu supplementation were observed on *Lactobacillus*, *Bacteroides* and *Enterobacteriaceae* counts*,* where higher Cu supplementation decreased these bacterial counts. Thus, Cu concentration from the basal diet should have been sufficient nutritionally according to NRC (1994), but microbiota groups may respond differently to Cu supplementation. Increasing dietary Cu non-selectively reduced the population of both beneficial bacteria i.e. *Lactobacillus* and pathogenic groups i.e. *Bacteroides* and *Enterobacteriaceae.* These results are similar to the work of Mei et al.^68^, where Cu supplementation decreased the counts of *Lactobacilli* and *Enterobacteriaceae* in the cecum of weaned piglets. According to De Boever et al.^69^, the high *Lactobacillus* population in the gut can impair lipid absorption as various *Lactobacillus* species have been reported to deconjugate bile acids in the gastrointestinal tract and consequently resulting in dietary energy losses. Copper supplementation at 200 mg/kg from CH inhibited the growth of *Bacteroides* and *Enterobacteriaceae* compared with the NC diet and 200 mg/kg CuSO_4_ supplemented diet. The CH supplemented diets compared to CuSO_4_, should have had a higher concentration of free ionic Cu–Cu ions not bind to other antagonistic nutrients—at lower parts of the intestine, which is soluble and can penetrate into the bacterial cells and produce hydrogen peroxide by changing the enzyme activity of the bacteria, leading to bacterial death^70^.

Short-chain fatty acids (the end-product of bacterial fermentation) play an essential role in gut physiology. It has been reported that SCFAs stimulate enterocyte growth and proliferation of enterocytes, which may partially explain the stimulating effect of the gut microbiome on intestinal growth^71^. Dietary Cu supplementation did not alter SCFAs production in the cecum of broilers in this study, except for a tendency of quadratic increase of propionic acid in response to increasing dietary Cu. Possibly the changing trends in bacterial populations were not of such a magnitude to affect SCFAs production. Along with the observed changes in villus height in the jejunum, it’s worth noting that the alteration of the intestinal morphology is possibly a direct effect of the dietary treatments, and partly an indirect effect through the changes of gut microbiota under conditions of this study.

A single layer of columnar epithelial cells that are connected by the tight junctions acts as the first protection against invasion of potentially harmful microorganisms, antigens and toxins from the intestinal lumen^72^. Reduced tight junction integrity and epithelial damage can thus result in a ‘leaky gut condition’ and are likely the main drivers for gut wall morphology changes, inflammation, systemic infection and malabsorption^73^. In the present study, the gene expression of the selected jejunal tight junction proteins was not influenced by the dietary Cu supplementation from either CuSO_4_ or CH. Similarly, the FITC-d quantities recovered in the serum were not affected by the dietary treatments, suggesting no occurrence of leaky gut. These results indicate that neither the NC diet was severely deficient in Cu, nor the high level of Cu supplementation up to 200 mg/kg may negatively affect the gut permeability in broiler chickens.

## Conclusion

In summary, the findings of the current study suggest that supplementation of broiler chicken’s diet with copper at growth-promoting levels (up to 200 mg/kg of finished feed) alters gut microbiota composition, does not negatively affect tissue oxidation and gut integrity of the bird. The supplementation of copper from copper hydroxychloride could be beneficial over copper sulfate in improving intestinal morphology and reducing copper excretion, especially at 200 mg/kg Cu as copper hydroxychloride. In addition, these results showed that a wheat-soybean based diet without any supplemental copper does not compromise liver health, intestinal morphology, and integrity.

## Data Availability

The datasets generated during and/or analyzed during the current study are available from the corresponding author on reasonable request.
